# Diurnal activity patterns and habitat use of juvenile *Pastinachus ater* in a coral reef flat environment

**DOI:** 10.1371/journal.pone.0228280

**Published:** 2020-02-27

**Authors:** A. P. B. Martins, M. R. Heupel, S. L. Bierwagen, A Chin, C Simpfendorfer

**Affiliations:** 1 Centre for Sustainable Tropical Fisheries and Aquaculture, College of Science and Engineering, James Cook University, Townsville, Australia; 2 Australian Institute of Marine Science, Townsville, Australia; Institut de recherche pour le developpement, FRANCE

## Abstract

Stingrays are thought to play important ecological roles in coral reef ecosystems. However, little is known about juvenile stingray movement patterns and habitat use in coral reefs. This study used active acoustic telemetry to determine fine-scale diel movement patterns and habitat use of juvenile cowtail stingrays (*Pastinachus ater*) in a coral reef flat environment. Seven cowtail stingrays (4 males and 3 females) were manually tracked between April and December 2016. Each individual was tracked over 2 days, generating a total of 14 active tracks ranging from 4.91 to 9 h. Specimens moved at an average speed of 2.44 m min^-1^ ± 0.87 SE, with minimum distances travelled ranging from 546 to 1446 m. Tracking data showed that juvenile cowtail stingrays move in response to tidal cycles, moving faster and in straighter pathways during incoming and outgoing tides. Juvenile cowtail stingrays also showed a strong affinity to sand flat areas and mangrove edge areas. These areas provide food resources and potential refuges for juvenile rays to avoid predators. Coral reef flats were identified as secondary refuge for juveniles during the lowest tides. Future research is necessary to fully unveil the major drivers of juvenile cowtail stingray seasonal and ontogenetic movement patterns and habitat use within coral reef flat environments. This information is important to establish a full understanding of juvenile cowtail stingray ecology, but could also improve management and conservation policies.

## Introduction

Stingrays (family Dasyatidae) are a diverse and widespread group of elasmobranchs. They can be found in a variety of habitats across the globe [1], including coral reefs. In these ecosystems, stingrays are thought to play important ecological roles, such as connecting trophic webs across habitats, enhancing nutrient recycling and energy flows, and controlling prey populations via predation and/or physical disturbances of soft-bottom microhabitats [2–6]. Stingrays are also an important fishing resource in many parts of the world [7–9], especially in coastal areas of developing countries where fish represent a significant portion of local food intake [10]. Stingray populations are facing elevated risks of extinction based on population declines caused by fishing and habitat loss [8].

Currently, some aspects of stingray biology are reasonably well understood, such as reproduction, diet, age and growth [11–16]. Nevertheless, little is known about the movement patterns and habitat use of stingrays inhabiting coral reefs [17, 18]–even less so about juvenile dasyatid rays. This lack of knowledge is a concern because, generally, survival during early life stages is essential for the health and persistence of slow growing, late-maturing and low fecundity species with a strong relationship between recruitment and population size, such as stingrays [19, 20]. Understanding the range and regularity of juvenile stingray movements and their direct relationships with the use of reef ecosystems may be beneficial in determining their dependence on reef habitats, their ecological role within these areas [21], and their response to anthropogenic threats [22].

Juvenile stingrays are often found in shallow soft bottom microhabitats [18, 23, 24]. While adults can feed in a wider variety of benthic habitats and move over deeper areas in search for food, juvenile distribution is often limited to these shallow soft bottom microhabitats where encounters with larger predators are reduced [25, 26] and feeding opportunities are increased [27].

Acoustic telemetry has been shown to be a successful tool to assess the activity patterns of both sharks and rays in coral reef habitats [21, 28]. However, the use of passive acoustic telemetry in areas juvenile stingrays often inhabit–shallow and turbid waters, and intertidal zones–is compromised by limitations such as shallow depth, habitat complexity, and water flow that affect detection ranges [29, 30]. Active telemetry is an effective alternative in such situations (in some cases the only feasible option) that can provide detailed movement information in habitats where physical attributes hinder the use of most telemetry methods [21, 31]. Thus, the broad aim of this work was to use active acoustic telemetry to determine fine-scale diel movement patterns and habitat use of juveniles of a stingray species known to commonly use coral reef systems–the cowtail stingray, *Pastinachus ater*.

## Materials and methods

### Study area

**Fig 1:**
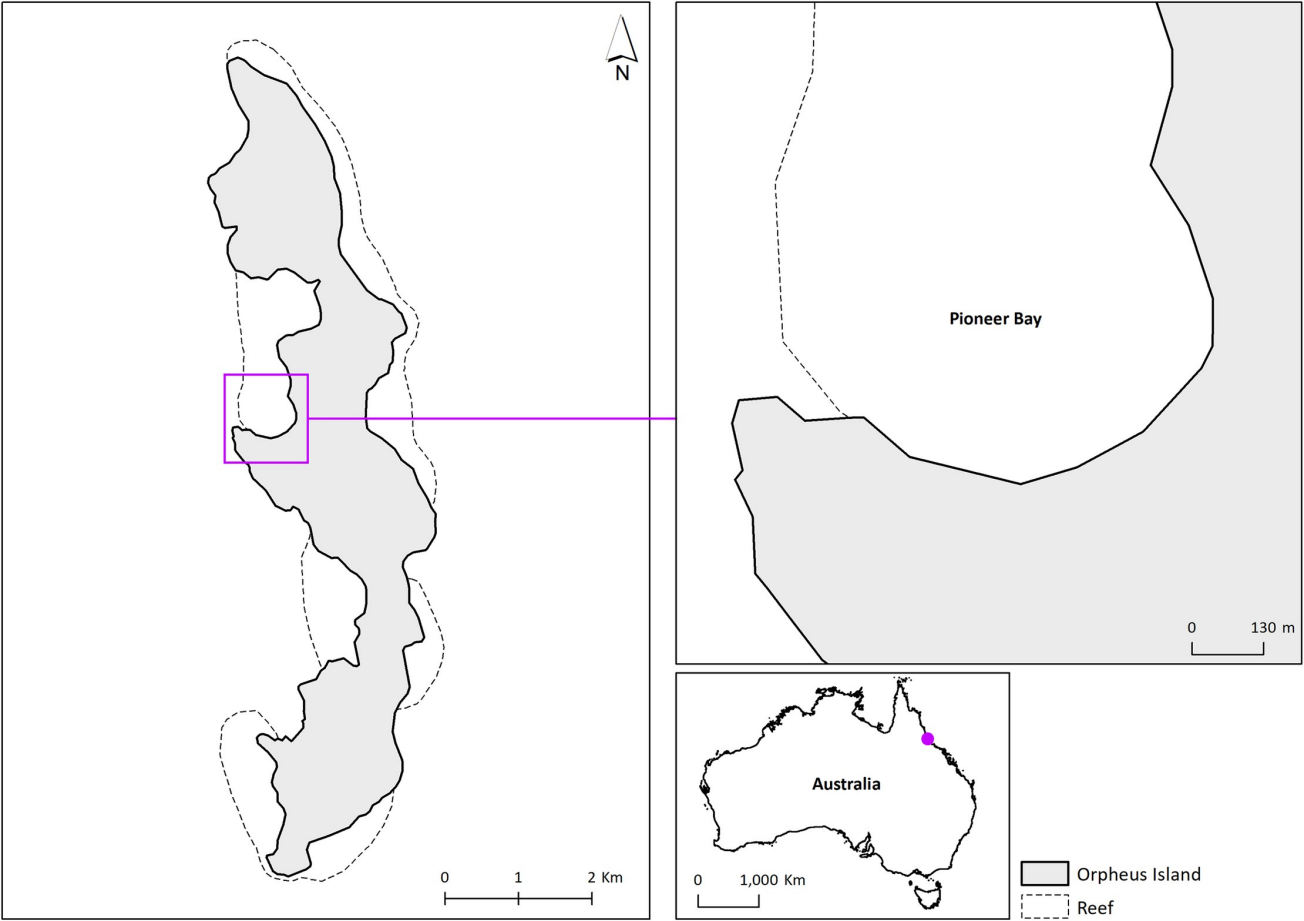
Map of Orpheus Island, Queensland, Australia.

**Fig 2:**
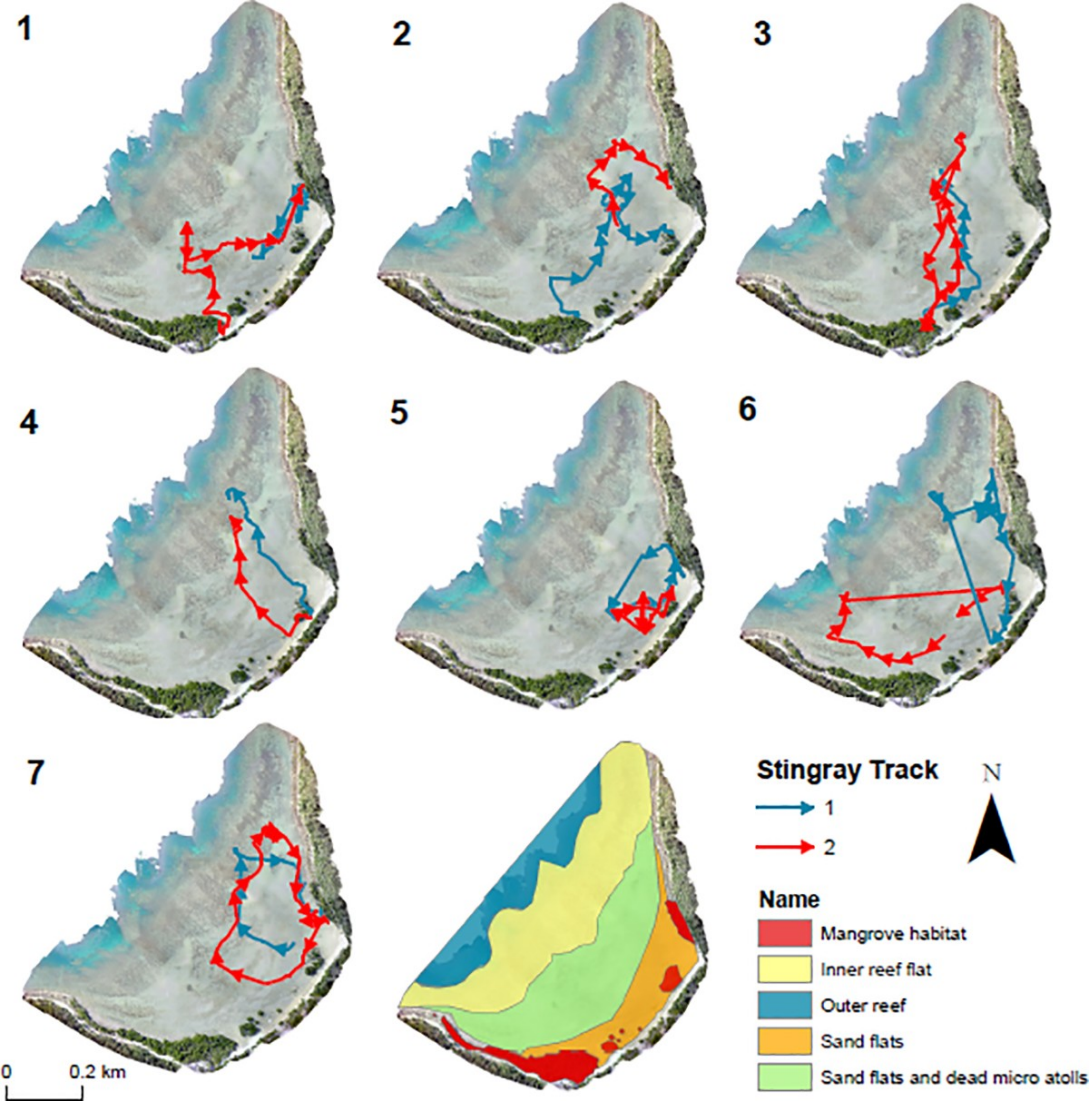
Tracks of juvenile cowtail stingrays and habitat types in Pioneer Bay. Each map represents two tracks of the same individual. Different styles of movement: (1) across the bay; (3) returning to the same area; (4) direct movements towards the reef flat; (6) use of northwest portion of the bay by one individual.

Pioneer Bay is located on the western side of Orpheus Island, in the Central Region of the Great Barrier Reef, Australia (18.6161° S, 146.4972° E; Fig 1). This 400-m wide bay has a reef flat and live coral areas influenced by semi-diurnal tidal variation. Mangrove patches (red mangrove, *Rhizophora mangle*; white mangrove, *Avicennia marina*; and myrtle mangrove, *Osbornia octodonta*) occur along the cemented beach deposits, especially in the southern flat area (Fig 2).

### Study species and capture methods

The cowtail stingray, *Pastinachus ater*, is a widespread species in the Indo–West Pacific. It inhabits intertidal lagoons, reef flats, reef slopes, bays and estuarine habitats. Individuals are born at approximately 18 cm disc width (DW) and can reach ~200 cm DW in full maturity [32]. Detailed maturity features for cowtail stingrays, such as length at first maturity (Lm), are unknown. Thus, only an estimate of male maturity can be determined by examining clasper calcification.

Cowtail stingrays were captured in the shallow waters of Pioneer Bay during outgoing tides using seine and dip nets between April and December 2016. Each animal was manually immobilized on a recreational paddle board for measurements (sex and disc width) and attachment of individually numbered spiracle tags. A Vemco V9 acoustic transmitter (21 mm in length, 1.6 g in water) was then attached to the spiracle tag of each juvenile stingray. Date, time and location (latitude and longitude) of the capture and release sites were also recorded. Handling procedures took less than 5 minutes.

Each Vemco V9 acoustic transmitter operated at a specific frequency (81, 78 or 75 KHz). Due to the short duration of the tracks, transmitters were set to continuously emit one acoustic signal per second, resulting in an estimated battery life of 5–7 days. Mirroring Davy et al. [30] tracking protocol, stingrays were released at their site of capture and actively tracked on foot both visually and with a hand held directional hydrophone (Vemco VH110) connected to an ultrasonic acoustic receiver (Vemco VR100). The location of the animal was recorded with a hand-held GPS every 5 minutes. Observers maintained a pre-established distance of 10 m from the tagged animal–minimising any potential impact on stingray natural movements yet remaining within the location error associated with the hand-held GPS. Each stingray was continually tracked for two days only during daylight hours. All tracks started at the outgoing tide and were completed during the incoming tide, when the water reached the mangrove patches or when natural light was no longer available. Depth, habitat type and behaviour of tagged stingrays were also recorded throughout the track. Due to shallow and clear water conditions during the study period, behaviours were visually identified and defined into three categories: searching (fast and circular movements), foraging (attempts to capture preys with high suspension of sediments and formation of feeding pits) and resting (moderate suspension of sediments followed by absence of movement). After two tracks, each animal was recaptured and the acoustic tag was removed.

Tidal stages were defined as: High tide– 1 h before to 1 h after the highest tidal height of a tidal cycle; Low tide– 1 h before to 1 h after the lowest tidal height of a tidal cycle; Incoming tide–period of increasing tide height between low and high tides; Outgoing tide–period of decreasing tide height between high and low tides.

This study was conducted under Great Barrier Reef Marine Park Authority permit G15/39987.1 and James Cook University animal ethics permit A2310.

### Statistical analysis

Tracking data were analysed using the adehabitatLT package in R [33]. Initially, rate of movement (ROM)–defined as the distance travelled by a specimen within 5 minutes–was calculated in m min^-1^ for all tracks. To identify the importance of the different habitats within Pioneer Bay for juvenile cowtail stingrays, 95% and 50% kernel utilisation distributions (KUD) were estimated using the package adehabitatHR [33]–with ad hoc method as the smoothing parameter. Based on KUD estimations, an Index of Reuse (IOR) was applied to evaluate if individual’s used the same areas during the two tracks, with higher values indicating congruence of daily areas. The method of Lavielle [33], a function used to estimate the number of segments building a track, was applied to fragment each track into intervals of specific movement patterns. Segments were plotted against tidal heights to graphically show possible variations in movement patterns. Tortuosity of movements–the degree of straightness of each track (ranging from 0 to 1, where zero indicates non-linear movement and one indicates linear movement)–was evaluated using an index of linearity (LI). The index was calculated iteratively for every 5 adjacent location points. To understand the effects of tidal heights and sex on ROM and LI, second order polynomial natural splines were applied using the R package glmmTMB [34] with individual as a random factor. ROM values were square-root transformed before analysis to achieve normality. Second order polynomial natural splines were also applied to evaluate a possible relationship between the maximum recorded distance from shore, defined as the point in time the stingray was furthest from shore (using a set shoreline across all measurements), and tidal height at low tides. The tracks were then placed into ArcGIS® to produce a map of cowtail stingray movement patterns and habitat use at Pioneer Bay.

## Results

Seven cowtail stingrays (4 males and 3 females) were manually tracked between April and December 2016 (Table 1). Disc width of individuals varied from 29 cm to 50 cm, with a mean of 39.5 cm. All male specimens were sexually immature. Due to the lack of external features to assess maturity levels, females were also considered sexually immature based on their similar DW to the males and the known use of Pioneer Bay as a nursery ground for other stingray species (30). Each individual was tracked over 2 days, generating a total of 14 active tracks ranging from 4.91 to 9 h. Specimens moved at an average speed of 2.44 m min^-1^ ± 0.87 SE, with minimum distances travelled ranging from 546 to 1446 m (Fig 2).

**Table 1:** Details of manual tracking events for juvenile cowtail stingrays at Orpheus Island. DW, disc width; ROM, rate of movement; LI, linearity; KUD, kernel utilization distribution; IOR, index of reuse.

Stingray	Sex	DW	Track	Date	Duration of track (h)	Min. dist. Travelled (m)	ROM (m min^-1^)	LI	KUD 95% (ha)	KUD 50% (ha)	IOR 95% (ha)	IOR 50% (ha)
1	M	37	1	1/04/2016	8	903.57	1.94	0.57	4.94	1.13		
			2	2/04/2016	9	1147.53	2.17	0.67	14.66	3.76	4.82	0
2	F	42	1	2/04/2016	7.83	1174.69	2.55	0.62	15.77	3.73		
			2	4/04/2016	5.33	585.20	1.77	0.59	6.25	1.53	4,42	0
3	F	33	1	5/04/2016	4.91	602.21	2.40	0.56	11.18	1.79		
			2	6/04/2016	6.91	1013.25	3.44	0.67	27.20	6.33	11.17	1.53
4	M	50	1	9/04/2016	5.58	546.28	1.65	0.72	11.83	3.17		
			2	10/04/2016	6.75	594.90	1.65	0.62	11.03	2.63	7.83	1.12
5	M	34	1	11/04/2016	8	672.27	1.49	0.61	4.89	0.87		
			2	12/04/2016	8.16	788.02	1.65	0.67	2.88	0.71	2.17	0.02
6	M	50	1	9/12/2016	5.75	1423.08	4.24	0.60	14.80	2.90		
			2	11/12/2016	5.08	884.64	2.90	0.65	19.77	3.27	3.89	0
7	F	29	1	13/12/2016	5.75	834.15	2.41	0.77	11.17	3.25		
			2	14/12/2016	7	1446.35	3.85	0.71	18.98	5.02	10.76	2.37

**Fig 3:**
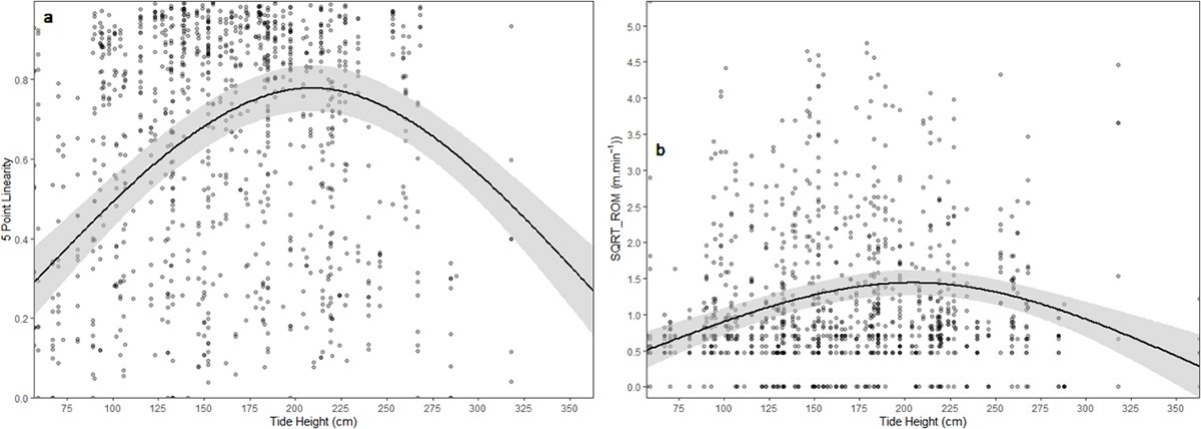
Effects of tide height on (a) LI and (b) ROM of juvenile cowtail stingrays. 95% confidence intervals are represented by the grey area.

All individuals remained in Pioneer Bay while tracked–suggesting juveniles have extended residencies within the bay. In fact, in four tracks, individuals returned to within < 100 m of their capture point on the next incoming tide. Despite the range of tidal heights in which tracks took place (57–363 cm), none of the individuals were observed in depths > 1 m (average depth: 30 cm). Both LI (p < 0.0001) and ROM (p < 0.0001) were found to be influenced by tidal height (Fig 3).

During outgoing tides, when water levels dropped and most of the reef flat became exposed, individuals moved toward the reef crest or outer crest margin. During this period, rays exhibited more direct and active movements (ROM = 3.27 m min^-1^ ± 2.44 SE; LI = 0.725 ± 0.06 SE) and were often observed in an intense searching mode and/or feeding in shallow sand flat areas (11 searching and 23 feeding events recorded). During incoming tides, rays used shallow areas, cruising at the limit of the water line and moving into areas as soon as there was sufficient water depth (tide height > 160 cm; e.g. sand flats and mangroves). During these incoming tides, stingrays interspersed resting behaviour (waiting for more areas to be made available by the rising water level) with more directed movements toward the shoreline (ROM = 2.61 m min^-1^ ± 2.12 SE; LI = 0.689 ± 0.10 SE). Occasional searching behaviours were observed during the incoming tide. However, individuals were not observed feeding either at high or low tide during tracks.

Juvenile cowtail stingrays moved in less linear paths and showed lower rates of movements during high (ROM = 0.678 m min^-1^ ± 0.28 SE; LI = 0.339 ± 0.13 SE) and low tides (ROM = 1.49 m min^-1^ ± 0.96 SE; LI = 0.527 ± 0.14 SE). Both ROM and LI values did not differ significantly between sexes (p = 0.09; p = 0.65, respectively). At the lowest tidal heights, individuals were observed using sand patches on the edge/within the reef flat as resting points. Individuals covered themselves with sand and remained immobile until the tide started to move back in. The average depth in these areas was approximately 20 cm. At the peak of the high tide, cowtail stingrays were more commonly encountered resting at mangrove patch edges and sand flats. They were rarely observed penetrating deep into mangrove habitats.

**Fig 4:**
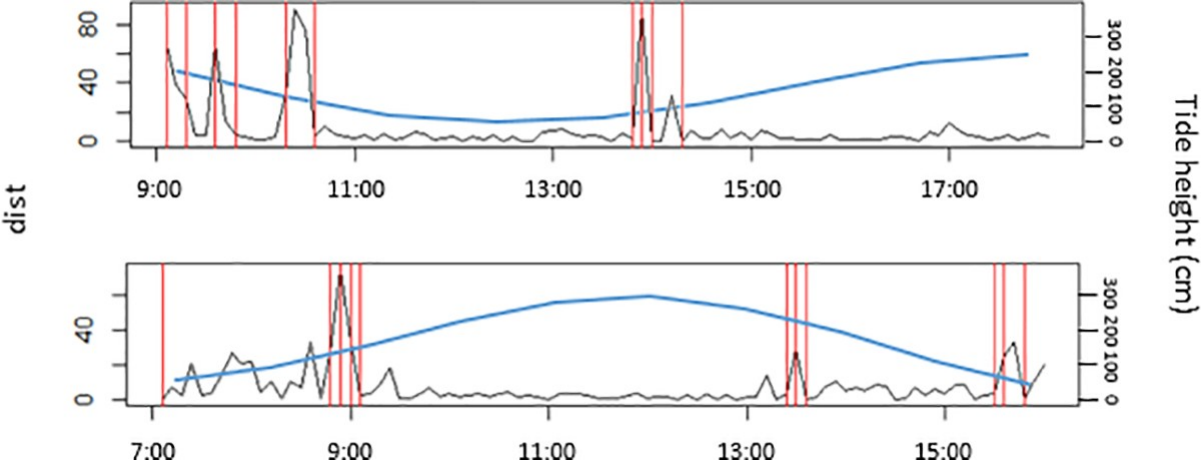
Lavielle method segmentation for two juvenile cowtail stingray active tracks in different tide heights. Black lines represent dist (distance between successive relocations), red lines indicate the boundaries of segments and the blue lines show the tide height.

**Fig 5:**
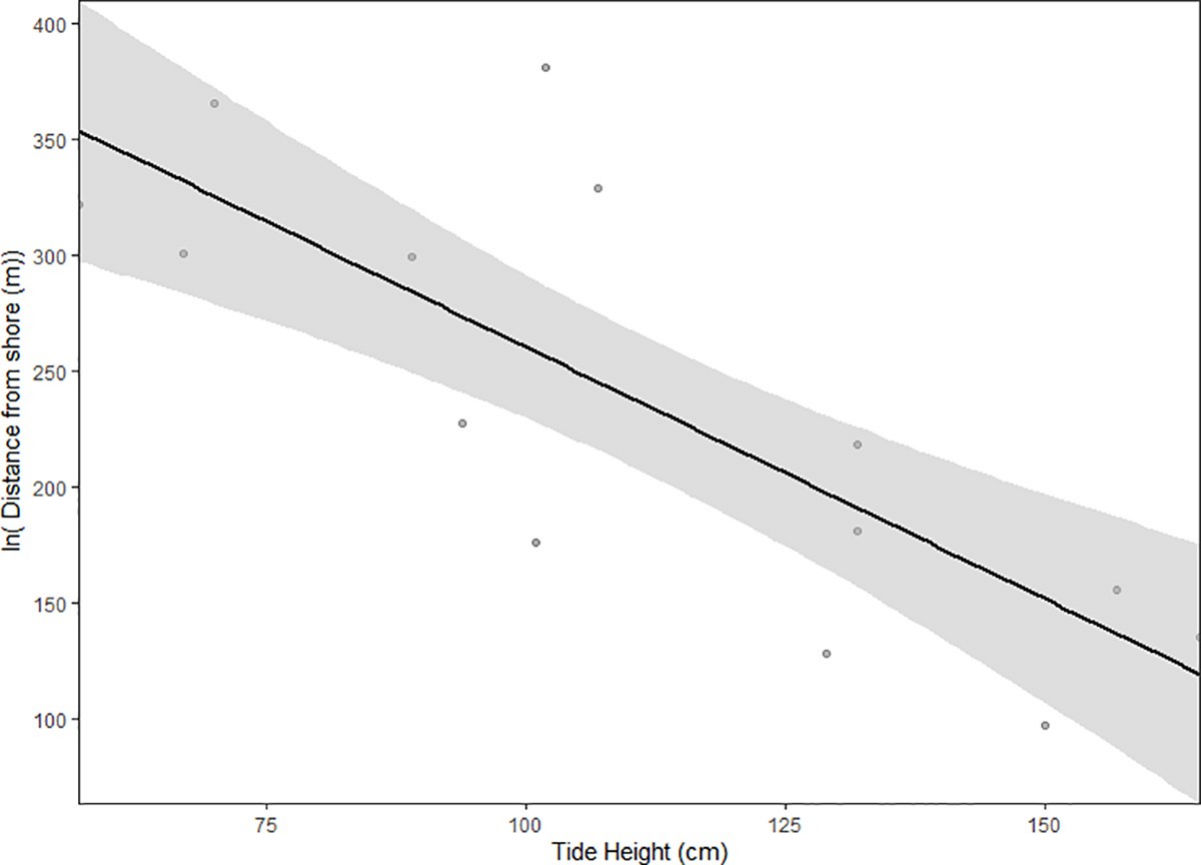
Relationship between the maximum recorded distance from shore and tide height at low tide. 95% confidence intervals are represented by the grey area.

**Fig 6:**
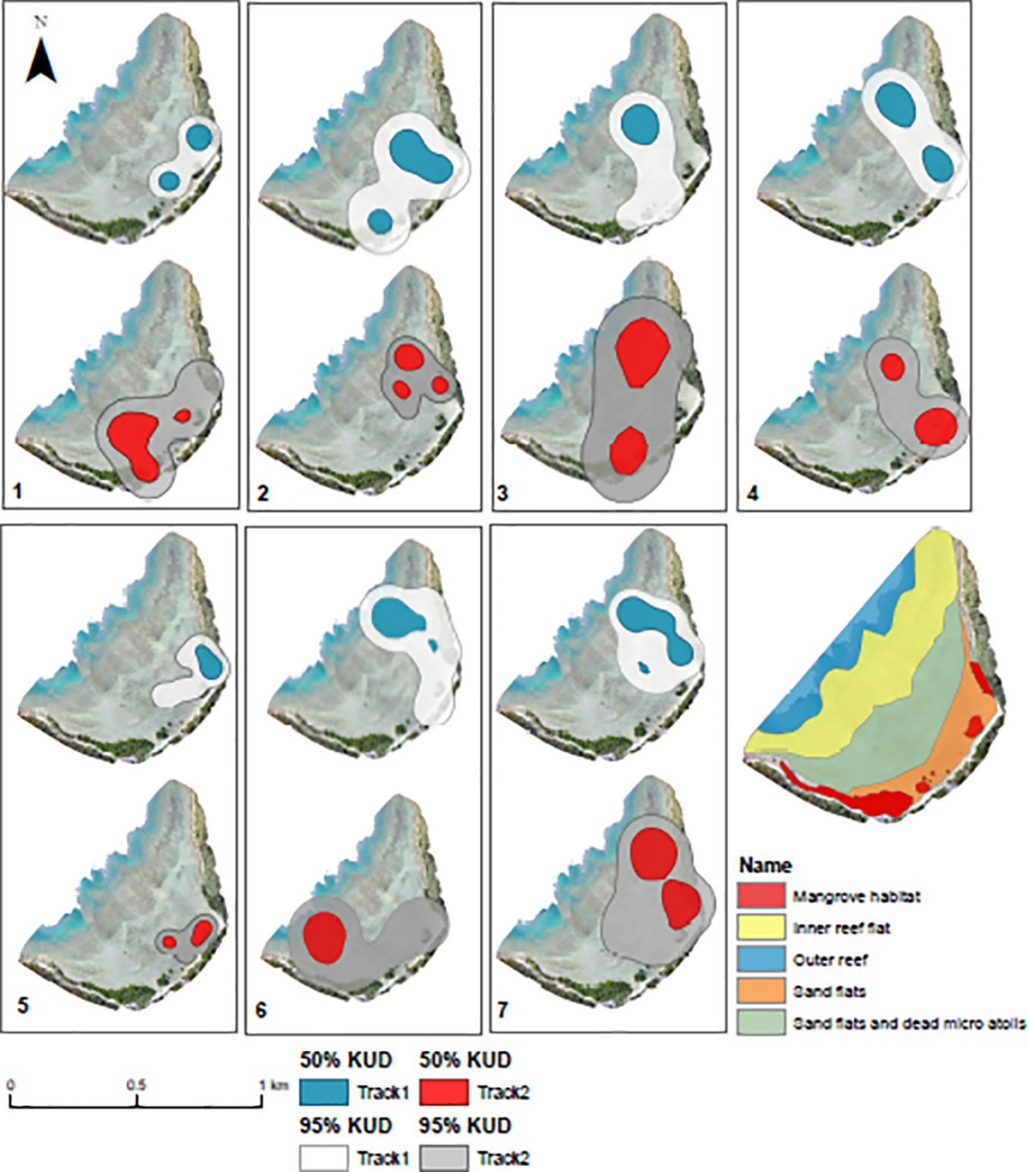
95% and 50% kernel utilisation distributions (KUD).

**Fig 7:**
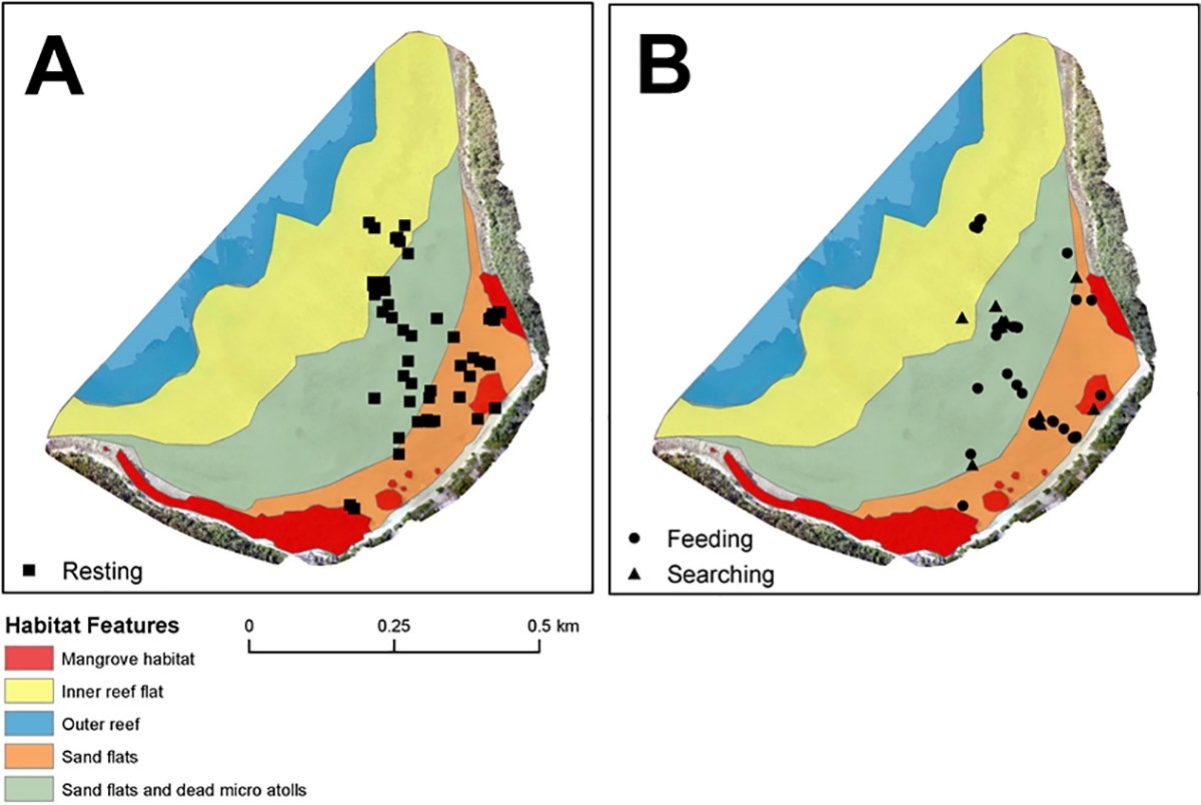
Location of (a) resting and (b) feeding/searching areas used by juvenile cowtail stingrays at Pioneer Bay.

The observed movement patterns of juvenile cowtail stingrays described above were reinforced by the Lavielle method. The number of segments of each track varied from 3 to 11 and tidal height influenced these activity patterns. Long periods of reduced activity (approximately 2–3 h) were interspersed by short sections of variable behaviour (< 60 min). Long segments in most tracks occurred during low and high tides, while short periods of variable behaviours mostly occurred during incoming or outgoing tides (Fig 4). The maximum distance from shore recorded for each individual was negatively related to the height of the tide (p < 0.0001; Fig 5), suggesting a wider tide variation throughout the day, with below average low tides, required individuals to move further toward the inner reef flat and outer reef to remain in shallow water. KUD results showed shallow sand flat areas located in the southeast portion of Pioneer Bay were intensively used by juvenile cowtail stingrays, constituting their primary habitat (Fig 6). Mangroves in the southern part of the bay were used when water depth was <1 m, with both feeding and resting behaviours observed in these areas in three instances (Fig 7). Areas of the western portion of the bay and outer reef were rarely utilized, likely due to the dense coral cover and lack of sand patches. IOR values ranged from 0.00 to 11.17 (Table 1). Stingrays 3, 4 and 7 showed the most restricted movements from all tracked individuals–reusing the same areas on each day of track.

## Discussion

The use of active acoustic telemetry showed that juvenile cowtail stingrays move in response to tidal cycles on an intertidal reef flat. Our results suggest that juveniles use shallow waters and/or camouflage techniques to avoid potential predators, and to access food resources in the sand flats. Juvenile cowtail stingrays spent the entire monitoring period within Pioneer Bay, with individuals not leaving the reef flat or reef crest for deeper waters during daylight hours. Although considered a major driver of stingray movement and habitat use in this and other studies (e.g., [35]), tidal variation did not appear to be as influential to juvenile cowtail stingray habitat use as they were for mangrove whiprays, *Urogymnus granulatus*, which were also tracked in Pioneer Bay [30]. According to Davy et al. [30], mangrove whiprays appear to reduce risk of predation by entering into mangrove habitats when the tide allows, residing within the complex structures of mangrove roots for as long as these structures were available. Juvenile mangrove whiprays only used sand flats when transiting to the fringing reef area–a secondary refuge during the lowest tides. In contrast, this study showed that juvenile cowtail stingrays were observed using mangrove habitats much less frequently even at higher tides, showing a stronger affinity to sand flat areas. This result is supported by video monitoring of mangrove areas by Kanno et al. [26].

Dasyatid species are known to commonly use sandy-bottom intertidal areas [12, 36, 37]. These areas can offer higher prey availability or better access to food resources [38–40]. In addition, juvenile cowtail stingray preference for shallow and soft sediment areas could reduce exposure to predators that cannot access these shallow waters [1, 22], and facilitate anti-predator behaviours, such as camouflage by burying and reduced movement rates [41]. In fact, large blacktip reef, *Carcharhinus melanopterus*, and lemon sharks, *Negaprion brevirostris*, are often seen in Pioneer Bay, with occasional predation attempts on juvenile stingray recorded (A Martins pers. obs.). Vaudo & Heithaus [24] also demonstrated that the activity space of three batoid species (*Glaucostegus typus*, *Himantura australis* and *Pateobatis fai*) was limited to nearshore and shallow waters of Shark Bay (Western Australia) likely as a result of predator avoidance.

Furthermore, as demonstrated by Papastamatiou et al. [42] and Espinoza et al. [43] for reef sharks, larger individuals often require a broad activity space in order to fulfill their energetic requirements. Stingray activity space in early life stages therefore might also be limited by low energy intake requirements. Thus, the limited movement and habitat use of juvenile cowtail stingrays within Pioneer Bay could be a trade-off between foraging opportunities, low energetic requirements and predator avoidance–while also reducing the direct competition for resources with co-occurring mangrove whiprays. Other strategies to avoid predation, such as aggregation behaviour, were not observed during the study period, but have been reported in other areas for cowtail stingrays [44].

ROM and LI were higher during the incoming and outgoing tides, with juvenile cowtail stingrays moving at faster rates and choosing more direct paths toward the reef flats or mangrove edge areas. Similar results were found by George et al. [45] for blacktip reef sharks in Pioneer Bay, suggesting this may be a common strategy for small, potentially vulnerable elasmobranchs in this area. Our results suggest that, during tidal changes, juvenile cowtail stingrays moved with a purpose and often changed their behaviour. By moving faster and using the shortest pathways, juvenile cowtail stingrays might reduce both their time in open/exposed areas during incoming tides and also the chance of getting trapped in shallow pools during outgoing tides. On the other hand, juvenile cowtail stingrays moved at a slower pace and chose non-linear paths during high and low tides. During these tidal phases, long periods of inactivity were observed, with individuals often adopting camouflage as a predator avoidance strategy. It’s worth noting that juvenile stingrays moved with the tide during the entire study period, regardless of tidal phase. Similar results were found by McInturf et al [46] for the broadnose sevengill sharks, *Notorynchus cepedianus*, in an estuarine habitat, with tracked individual movements mostly corresponding to current direction.

Although variations between sexes have been documented in shark and ray movement pattern studies [47], both ROM and LI values did not differ significantly between sexes in this study. Similar results have been found by Dale et al. [48] and Cerutti-Pereyra et al. [49], indicating that differences in movement patterns and habitat use between sexes do not occur for some batoid species at early life stages. The restricted activity space could also be a factor hindering sexual segregation or sex-specific behaviours. These differences may develop once individuals become sexually mature and have larger areas of available habitat. However, this study only involved a small number of individuals, so further studies are required to fully understand the potential for ontogenetic shifts in cowtail stingray behaviour and to confidently proclaim that the observed movement patterns and habitat use are consistent over time.

Different from the results found by Davy et al. [30] for mangrove whiprays, juvenile cowtail stingrays were observed feeding more often during the outgoing tide. In addition, juvenile cowtail stingrays were not detected feeding either at high or low tides. Results also showed individual feeding activities were mostly restricted to the soft-bottom area located on the southeast portion of Pioneer Bay. According to Ajemian & Powers [50], the use of specific patches for foraging purposes is often related to the availability of resources. Unfortunately, information on cowtail stingray diet and prey availability in Pioneer Bay are not available. However, several studies on bottom dwelling batoid species have shown their preferences for benthic invertebrates, such as annelids, crustaceans, molluscs, bivalves and gastropods [39, 40]–all of which are commonly found across the sand flats of Pioneer Bay (A Martins pers. obs.). In this way, it may be that mangrove whiprays and cowtail stingrays target similar infauna in Pioneer Bay and partition habitats spatially rather than temporally, as suggested for some reef shark species [51].

## Conclusion

In this study, the use of active acoustic telemetry has helped to provide the first detailed examination of the spatial ecology of cowtail stingrays in a coral reef flat environment. Results have shown juvenile cowtail stingray preferences for sand flat and mangrove edge habitats within Pioneer Bay. These areas provide food resources and the ability to more successfully avoid potential predators. Coral reef flats were identified as secondary refuge for juvenile cowtail stingrays during the lowest tides. In addition, juvenile cowtail stingray movements were shown to be strongly guided by tidal cycles. These findings highlight the need to better comprehend the movement patterns and habitat use of juvenile cowtail stingrays at different spatial and temporal scales. Future research is necessary to fully unveil the major drivers involved in juvenile cowtail stingray seasonal and ontogenetic movement patterns and habitat use within coral reef flat environments. This information is important to establish a full understanding of juvenile cowtail stingray ecology, but also could support management and conservation policies.
